# *Naegleria fowleri* and Risk of Primary Amoebic Meningoencephalitis in a Changing Climate: A Scoping Review of Biomedical Literature

**DOI:** 10.3390/ijerph23060764

**Published:** 2026-06-06

**Authors:** Janette DeFelice

**Affiliations:** Department of Health Sciences, DePaul University, Chicago, IL 60614, USA; jdefelic@depaul.edu

**Keywords:** *Naegleria fowleri*, primary amoebic meningoencephalitis, climate change, waterborne disease, public health preparedness, water quality

## Abstract

**Highlights:**

**Public health relevance—How does this work relate to a public health issue?**

- *Naegleria fowleri* is a thermophilic amoeba that causes a nearly always fatal central nervous system infection called primary amoebic meningoencephalitis (PAM).
- Climate change may impact the viability, biotic environment, and/or geographic range of *N. fowleri,* potentially putting more humans at risk of contracting PAM.

**Public health significance—Why is this work of significance to public health?**

- This review synthesizes the peer-reviewed literature regarding the risk of disease caused by *N. fowleri*, specifically in the context of climate change, and identifies knowledge gaps regarding environmental, behavioral, biological/clinical, and infrastructural risk dimensions.

**Public health implications—What are the key implications or messages for practitioners, policy makers and/or researchers in public health?**

- Public health preparedness should prioritize primary prevention through environmental surveillance and determination of the geographic range of *N. fowleri*, and secondary prevention through increasing clinician awareness in order to improve case detection and quantify true disease burden.

**Abstract:**

**Objective:** *Naegleria fowleri*, known as the brain-eating amoeba, is a thermophilic, freshwater amoeba causing primary amoebic meningoencephalitis (PAM), a disease that progresses rapidly from symptom onset to death. Climate change is causing surface water temperatures to increase, providing a hospitable environment for *N. fowleri*, possibly increasing risk factors for PAM. This review synthesizes the peer-reviewed biomedical literature published between January 2012 and December 2025, examining the risk of *N. fowleri* infection in the context of a warming climate. **Methods:** A scoping review was conducted searching PubMed, Scopus, and Environment Complete. Data were extracted using a structured coding framework, and risk dimensions were derived inductively during the coding process. **Results:** Twenty-seven articles met inclusion criteria. Analysis revealed four dimensions of risk (environmental, behavioral, clinical/biological, and infrastructural). The environmental risk dimension highlighted gaps in understanding geographic range expansion and organism ecology. The behavioral dimension identified recreational water exposure, sinus rinsing, and travel as primary risk drivers. The clinical/biological dimension highlighted the need for standards and capacity in diagnosis and treatment, as well as research into pathogenicity. The infrastructural dimension identified gaps in water distribution system surveillance and disinfectant efficacy at high temperatures. **Discussion:** This review maps environmental, behavioral, clinical/biological, and infrastructural dimensions of *N. fowleri* disease risk onto a hazard/exposure/vulnerability framework, highlighting major gaps surrounding exposure and vulnerability. Uncertainties remain in hazard habitat favorability factors, human behavior, and water distribution systems. Emphasis should be placed on characterizing the *hazard* through environmental testing and determining geographic range, and addressing *vulnerability* by increasing clinician awareness, which serves double duty in both initiating early empiric treatment and efforts to quantify true disease burden.

## 1. Introduction

*Naegleria fowleri*, more commonly known as the brain-eating amoeba, is a free-living, thermophilic, freshwater amoeba that is known to cause a nearly always fatal central nervous system infection called primary amoebic meningoencephalitis (PAM), a disease that progresses rapidly (days to weeks) from symptom onset to death. Although PAM is rare, there is concern that climate change and its consequent warming of freshwater sources may impact the viability, biotic environment, and/or geographic range of *N. fowleri*, potentially putting more humans at risk of contracting PAM.

*N. fowleri* thrives at water temperatures ranging from 30 to 46 °C [1]. It has been found on all continents except for Antarctica and as far north as Connecticut, Illinois, and Minnesota in the United States [2,3,4,5]. The amoeba has not only been found in lakes but also in drinking water (tap, well, and public distribution systems), as well as in rainwater catchment tanks and swimming pools [6,7,8,9,10,11]. Furthermore, a 2020 systematic review with meta-analysis of over 100 studies found evidence of widespread global prevalence of *Naegleria* species (with *N. fowleri* accounting for 23.27%) across multiple water sources [12]. The organism can be found protected by a biofilm, but it is susceptible to adequate levels of chlorination [13].

*N. fowleri* has three distinct life phases: trophozoite, flagellate, and cyst. The trophozoite form is the infectious form of the organism. When motility is required, it converts to its flagellated stage. Additionally, when environmental conditions are not optimal for survival, it transforms to the cyst stage, a dormant state that confers greater resistance to adverse conditions. Because of these distinct life stages, the amoeba is capable of surviving through a wide range of temperatures [13].

*N. fowleri* causes the central nervous system (CNS) infection, Primary Amoebic Meningoencephalitis (PAM), in humans. Usually, in the trophozoite stage, the amoeba gains entry through the nasal passages and travels to the brain via the olfactory nerves. Patient history often includes swimming in a warm freshwater body of water, use of a neti pot for wellness-related sinus rinsing, or the cultural practice of nasal ablution (ritual sinus rinsing). Some cases have also been reported after visiting recreational water parks. Incubation time is three to eight days, with death occurring in seven to ten days of symptom onset [1].

More than 400 cases of PAM have been reported in the literature since 1965 [13]. As of 2024, there have been only eight survivors [14,15]. It is estimated that the mortality rate for PAM is approximately 95%. Important to note, PAM is thought to be underreported because it has been historically misdiagnosed as viral or bacterial meningitis and can only reliably be detected on post-mortem examination of the brain and meninges [1,13]. Early empiric treatment is a crucial intervention, as there is no routinely reliable diagnostic test.

In the United States, the incidence of PAM has historically followed a seasonal pattern, peaking in the warmer summer months, especially in the southern states [16]. It has been argued that rising global temperatures will contribute to expanding the global geographic range in which *N. fowleri* can thrive toward the poles [1,4,17]. In recent years, we have seen the habitat of *N. fowleri* expand to include midwestern and northern states, even causing multiple cases of PAM in Minnesota, where the air temperature was 3.6 °C above average for that time of year [4,14]. Though we have not seen increasing numbers of PAM cases in the United States, the numbers of recorded cases have increased worldwide since the year 2000 [1]. It remains unclear, though, whether this increase is due to a rise in incidence or to increased awareness and improved diagnostic capacity.

Because of historic challenges with diagnosis and subsequent underreporting of PAM, the true global burden of disease remains unknown. However, occurrence of infection outside of the amoeba’s expected geographic range raises concern about whether *N. fowleri* should be considered an emerging, climate-sensitive pathogen. As the habitat of *N. fowleri* continues to expand, and global average temperature continues to increase, a clearer understanding of the multiple dimensions of risk associated with infection becomes increasingly urgent.

The Sixth Assessment Report of the Intergovernmental Panel on Climate Change (IPCC AR6) provides us a framework for organizing multiple dimensions of climate risk: “in the context of climate change impacts, risks result from dynamic interactions between climate-related *hazards* with the *exposure* and *vulnerability* of the affected human or ecological system to the hazards.” [18]. In this case, the *hazard* comprises *N. fowleri*’s thermotolerant nature and changing environmental habitat in response to increasing water temperatures. The *exposure* is demonstrated by human behavioral patterns (especially in the context of a warming climate) and geographic proximity to affected water sources. *Vulnerability* emerges in the knowledge gaps concerning clinical awareness, diagnostic capacity, prevention, water infrastructure surveillance, and treatment. Applying the hazard/exposure/vulnerability framework to the biomedical literature is an effective lens through which to assess what is known and not known about the risks, and where knowledge gaps leave populations most vulnerable.

## 2. Rationale and Objectives

As global temperatures rise, freshwater bodies become increasingly hospitable habitats for *N. fowleri*. A potential link between climate change and the incidence and geographic range of infection with the organism and the public health implications warrant systematic examination. The aim of this scoping review is to map and synthesize the extent and nature of the peer-reviewed literature published between 2012 and 2025 regarding the risk of disease caused by *N. fowleri*, specifically in the context of climate-driven environmental changes such as rising temperatures and altered water ecology. This review seeks to identify existing knowledge (including dimensions of risk), highlight research gaps, and inform future directions for surveillance, prevention, and public health preparedness. The IPCC AR6 hazard/exposure/vulnerability framework (introduced above) is used to interpret findings and position them within the context of broader climate change risk assessment.

Specifically, this review aims to address the following:

1. What is the extent and nature of the peer-reviewed literature published between 2012 and 2025 discussing *N. fowleri* in the context of a warming climate?
2. How is risk characterized in that literature (i.e., what dimensions of risk emerge)?
3. Where do gaps exist in the literature in terms of addressing hazard, exposure, and vulnerability, and what are the implications for public health surveillance, prevention, and preparedness?

## 3. Methods

A literature search was conducted using the PubMed, Scopus, and Environment Complete databases for articles published from January 2012 to December 2025 which contain the search terms ‘naegleria fowleri AND climate change AND primary amoebic meningoencephalitis’ and ‘naegleria fowleri AND global warming AND primary amoebic meningoencephalitis.’ The year 2012 was specifically chosen as a starting point because there has been an increasing number of publications regarding *N. fowleri* in the context of climate change since that year. This review was not designed as a broad survey of the literature on *N. fowleri*. Rather, search terms were deliberately restricted to situate *N. fowleri* and PAM explicitly within the context of climate change as a risk factor, in order to gauge the extent to which *N. fowleri* is being framed as an emerging, climate change-related cause of disease. This review followed the Preferred Reporting Items for Systematic reviews and Meta-Analyses extension for Scoping Reviews (PRISMA-ScR) checklist, and is registered on the Open Science Framework, DOI: 10.17605/OSF.IO/UCQAS.

### 3.1. Eligibility Criteria

Inclusion criteria: all peer-reviewed articles with publication dates from January 2012 to December 2025 which contained the search terms above and also contained the term *Naegleria fowleri* in the title or abstract were moved to full-text review. Requiring *N. fowleri* to be in the title or abstract ensured the included articles would have a specific focus on the pathogen of interest. Exclusion criteria included: (a) book reviews, (b) letters to the editor or editorials, (c) commentaries, (d) book chapters, (e) non-English articles, (f) papers focused on other amoeba species, and (g) papers not relevant to discussion of risk. References of included articles were not hand-searched. Searches were last conducted on 29 April 2026.

### 3.2. Data Charting

Data collected from studies include bibliographic information (authors, date, journal), type of study, climate framing (direct, indirect), evidence type, key findings, gaps or recommendations identified by authors, and relevance for public health preparedness (diagnostic awareness, prevention, surveillance, communication, treatment). Data were extracted and analyzed by a single reviewer. Risk dimensions were derived inductively through iterative review of included studies. Related concepts were grouped into broader thematic categories, resulting in four dimensions of risk: environmental, behavioral, clinical/biological, and infrastructural.

## 4. Results

### 4.1. Study Selection

**Figure 1:**
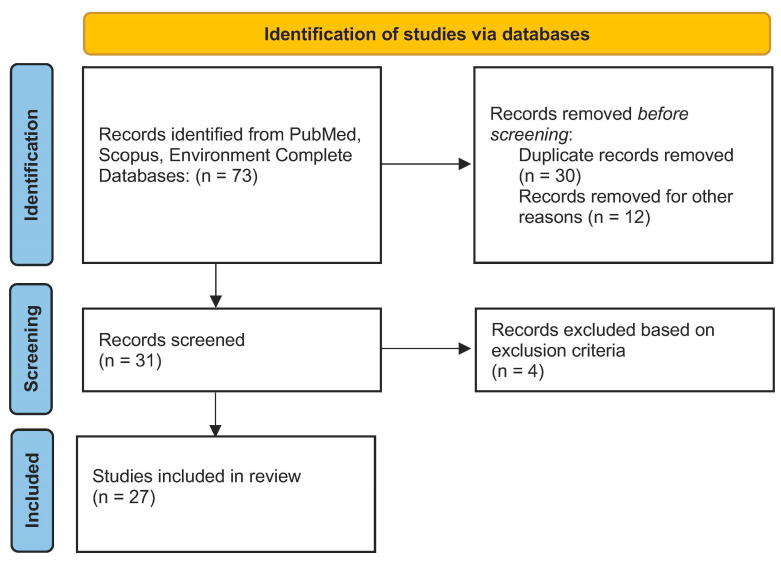
PRISMA Flow Diagram showing identification, screening, eligibility, and inclusion of studies examining *N. fowleri* and climate change from 2012 to 2025.

A total of 73 articles were found during the preliminary search of the three databases. From that, 30 duplicate articles were removed, and 12 articles were excluded based on title and abstract screening. This left 31 articles eligible for full-text screening, four of which were excluded based on exclusion criteria above. Ultimately, 27 articles were included in this review (See Figure 1).

### 4.2. Study Characteristics

Eighteen out of the 27 articles mentioned *Naegleria fowleri* in the title [1,4,13,19,20,21,22,23,24,25,26,27,28,29,30,31,32,33]. Five of the articles included ‘meningoencephalitis’ or ‘primary amoebic meningoencephalitis’ or ‘primary amebic meningoencephalitis’ in the title [16,17,20,27,29]. Two articles indirectly referred to climate change in the title [17,34]. None of the articles mentioned both *Naegleria fowleri* and climate change in the title. Twelve articles were review articles [1,13,16,17,25,28,29,32,34,35,36,37]. Eleven articles were original research [19,21,22,23,24,26,31,33,38,39,40]. Four articles were case reports [4,20,27,30].

### 4.3. Climate Change Framing

In total, nine articles addressed research questions directly within the context of climate change [1,4,17,25,28,31,34,37,40]. The other 18 did not, referring to the possibility of a warming climate affecting outcomes related to *N. fowleri* [13,16,19,20,21,22,23,24,26,27,29,30,32,33,35,36,38,39].

### 4.4. Dimensions of Risk

Risk dimensions were derived inductively during coding. They include: environmental risk, behavioral risk, clinical/biological risk, and infrastructural risk. Some articles address more than one risk dimension [1,4,17,20,24,25,28,29,37]. Each article is discussed under its dominant dimension; crossover to other dimensions is noted.

### 4.5. Environmental Risk Dimension

Nine articles were coded as primarily addressing environmental risk. Risk factors included viability, distribution, and abundance of *N. fowleri*; conditions that allow the organism to thrive; and evidence of expansion of geographic range. Viewed through the lens of the IPCC framework, this risk dimension corresponds most directly to the concept of *hazard*.

Maciver et al. claim that evidence of worldwide incidence of infection with *N. fowleri* has increased since the year 2000 [1]. However, they note that recorded incidence may be affected by factors such as increased knowledge of the disease PAM and sensationalization in the media of *N. fowleri* as the “brain-eating amoeba.” They provide evidence that worldwide, more males are affected than females. An assertion is made that, because of the summertime seasonality of PAM and the survivability of *N. fowleri* at temperatures in excess of 46 °C, warming global temperatures will expand the geographic range of the parasite and will cause a higher incidence of disease. (Also addresses behavioral and clinical dimensions; see below).

The authors ask several direct questions regarding gaps in knowledge that point to directions in further research: “What is the true burden of PAM worldwide? Is the incidence of PAM actually increasing or are we just more aware of it? Is the geographic distribution expanding and/or will it, due to climate change? Why do young males seem to be at more risk than other people? What factors dictate the distribution of *N. fowleri*? How will we diagnose cases more rapidly, and how can we treat these cases more effectively?”

Stahl et al. make multiple contributions to the environmental risk dimension. In their 2020 article, the authors assemble important abiotic and biotic factors that affect the ability of *N. fowleri* to thrive, and highlight areas where future research is needed [13]. They show that the parasite is highly thermotolerant and can survive temperatures in the 46 ° C range. Additionally, *N. fowleri* can survive in a wide pH range, from 2 to 8.15. The amoeba has low tolerance for salinity and is sensitive to “adequate” levels of chlorination; however, a biofilm can provide protection. They feed on bacteria and are preyed upon by other amoebae. According to the authors, more efficient detection and quantification methods are needed to gather more information on abiotic and biotic factors, as well as other factors, that affect the growth and viability of *N. fowleri* in order to mitigate the risk of disease in a warming climate.

Stahl et al. (2023) dive further into their previous work on biotic and abiotic factors that influence the abundance of *N. fowleri* by demonstrating that temperature and salinity of water interact to provide more or less hospitable environments for amoebal growth—lower salinity encouraged growth at all temperatures, while lower temperatures encouraged growth in high salinity [13,23]. The authors’ vision is that knowledge of ecological factors that influence the hardiness of *N. fowleri* may help prevent human infection in our changing climate.

In 2025, Stahl et al. used ecological niche modeling to project the suitability of *N. fowleri* freshwater habitats in the United States under present-day and future warming scenarios [31]. Results showed that “increases in habitat suitability for some geographic regions, especially in northern states, suggested a potential northward expansion of *N. fowleri*.” The authors call for further experimental research into the association between environmental factors and viability of the amoebae to improve ecological niche modeling.

Xue et al. demonstrate that *N. fowleri* can survive in the brackish water of Lake Pontchartrain at salinity levels from 0.1 to 2.37 ppt [24]. They emphasize that *N. fowleri*’s viability at these salinity levels should be considered a risk factor for those using brackish recreational waters. They detected the free-living amoebae in waters at temperatures ranging from 29 to 47 °C. Interestingly, they found a positive statistically significant correlation between *N. fowleri* and qPCR *E. coli* results, though this association was not present when *E. coli* was quantified by other methods. The authors suggest more research to focus on the role of sediment as a source of *N. fowleri*. (Also addresses infrastructural dimension; see below).

Leal dos Santos et al. consider *N. fowleri* abundance and infection within the framework of the One Health approach [25]. The One Health approach emphasizes the connection between human health, animal health, and the environment. This article uses the One Health framework to analyze what is known about *N. fowleri* and the connection of its increasing abundance to climate change. The authors state, “Our results indicate that climate change plays a major role in the growth and dispersion of the pathogen in the environment, causing damage to humans and animals.” The authors call for further investigation into how ecosystem changes lead to dispersion of the pathogen. (Also addresses behavioral and clinical dimensions; see below).

Dey et al. find free-living *Naegleria* species in recreational freshwater bodies in Alberta, Canada, providing evidence of northward expansion of species [40]. However, no *N. fowleri* were specifically confirmed. Despite this, the authors recommend ongoing freshwater surveillance and more research to further define distribution and microbial ecology.

Bright and Gerba review the natural occurrence of *N. fowleri* in groundwater and geothermal waters, reporting on several cases of PAM that have been linked to these sources [32]. They note significant gaps in understanding why the organism thrives in some water sources and not others, the role of biofilms, and possible poleward extension of habitat due to climate-driven warming.

In a case report with supporting environmental and climatological data, Kemble et al. present the case of a seven-year-old female in Minnesota who succumbed to PAM on day four of her illness, as confirmed by histopathological examination of brain tissue on autopsy [4]. Environmental samples from one of the three freshwater bodies in which she swam yielded *N. fowleri*. The striking thing about this case is that it did not occur in a southern state with a historically warm climate. The authors note that temperatures had been trending up in Minnesota over the prior 30 years, and that average daily minimum and maximum temperatures in the two weeks preceding the onset of the girl’s illness were higher than normal. The authors are careful to point out that the warmth of the water alone may not have been enough to encourage the growth of the amoeba. There were also indicators of poor water quality in the particular lake that yielded *N. fowleri*—“visible suspended organic matter and sediment and storm water drainage entering the lake.” [4]. The authors further suggest that other factors such as intra-amoebic bacteria may alter pathogenicity or host immune response. (Also addresses clinical/biological dimension; see below).

### 4.6. Behavioral Risk Dimension

Two articles were coded as primarily addressing behavioral risk. Risk factors included human activities that bring individuals into contact with *N. fowleri*, including recreational water exposure, sinus rinsing, and travel. Viewed through the lens of the IPCC framework, this risk dimension corresponds most directly to the concept of *exposure*.

Diaz presents a retrospective, longitudinal, descriptive and statistical analysis of 121 CDC-confirmed cases of PAM in the United States, demonstrating that the disease is most prevalent among young males with recreational water exposure in the southern states [16]. The author also found there were more cases of PAM in the 30-year reporting period from 1977 to 2007 than were in an earlier 40-year reporting period from 1937 to 1976 and more clusters of four or more cases of PAM in the later reporting period. The article emphasizes prevention through education and behavior modification: avoiding water-related activities in bodies of warm freshwater, avoiding water entering the nose, avoiding stirring up sediment, and using properly treated water in neti pots for sinus rinsing. A call is made for more study into the increased risk in a warming climate.

In another case report, Hong et al. recount the case of a 52-year-old male who returned to South Korea after an extended stay in Thailand and died on day 13 after symptom onset [20]. Polymerase-chain reaction testing of cerebrospinal fluid isolates revealed a *N. fowleri* strain that was 99.64% similar to a strain that had been isolated from the CSF of a Norwegian man who had also traveled to Thailand. The authors call for heightened vigilance among healthcare practitioners for this disease because of its rapid progression, especially in patients who may have been traveling to warmer regions. (Also addresses clinical dimension; see below).

### 4.7. Clinical/Biological Risk Dimension

Fourteen articles were coded as primarily addressing clinical/biological risk. Risk factors included gaps in diagnostic capacity, clinical knowledge, and treatment. Further gaps are identified in knowledge of pathogenicity, possible virulence factors, and changes in molecular structure that increase infectivity. Viewed through the lens of the IPCC framework, this risk dimension corresponds most directly to the concept of *vulnerability*.

Cooper et al. summarize treatment plans recommended for patients with PAM, a disease which they assert, due to increasing global temperatures and warming waters providing a hospitable environment for the proliferation of *N. fowleri*, should be on every clinician’s differential diagnosis for meningitis “regardless of the latitude.” [17] (Also addresses environmental dimension; see above).

Rizo-Liendo et al. show that statins may have promise as a chemotherapeutic adjunct in treating patients with PAM due to the inhibition of 3-hidroxy-3-methylglutaryl coenzyme A reductase, an enzyme essential to the synthesis of *N. fowleri*’s cell membrane [21]. The authors call for further studies into the efficacy of statins, specifically fluvastatin, as a therapeutic agent against PAM.

In 2022, Siddiqui first-authored three articles showing that certain plant metabolites, as well as imidazothiazole derivatives (which had previously been demonstrated to have anti-tumor as well as anti-microbial effects), and zinc oxide nanoconjugates (which had previously been demonstrated to have antibacterial and amoebicidal effects) were effective against *N. fowleri*, while being minimally cytotoxic to human cells in vitro [22,38,39]. Further research is suggested to test for the efficacy of the above possible therapeutic compounds against the cyst form.

Mungroo et al. and Zhang et al. both review features such as the epidemiology, management, and pathogenesis of various and opportunistic amoebal pathogens, including *N. fowleri* [35,36]. The authors suggest a need for expanding in vitro research into viable therapies, more research into metal-conjugated nanoparticles and intra-nasal drug delivery for therapy, and an urgent need for interdisciplinary research collaborations between academia, the pharmaceutical industry, and water distribution utilities, as well as standards for detection and treatment of PAM.

Herman et al. discuss a novel understanding of the biology and infectivity of *N. fowleri* at the cellular systems level by sequencing the genomes of two *N. fowleri* strains and analyzing differing levels of pathogenicity in a third strain, but do not address how, or if, temperature may affect this [19].

Kou et al. report a case in which metagenomic next-generation sequencing (mNGS), targeted PCR, and microscopic analysis of CSF allowed for rapid molecular diagnosis in an individual infected with *N. fowleri* [27]. The authors suggest that clinicians should initiate anti-amoebic therapy empirically in patients presenting with history consistent with a PAM diagnosis. Further, it is suggested that public health efforts should focus on monitoring and disinfecting recreational water facilities and alerting the public when the risk of contracting PAM is increased.

Phung et al. present the case of PAM in a 10-month-old in Vietnam with no reported direct exposure to untreated freshwater [30]. The diagnosis was made via multiplex real-time polymerase chain reaction (PCR). The authors note the particular vulnerability in under-resourced areas that may not have access to advanced diagnostic tools.

Hall et al. review the characteristics and treatment courses of five PAM survivors [29]. The authors purport that diagnosis is largely dependent on the patient’s history of exposure, physician’s high index of suspicion, and CSF studies. They also point to the gap in experimental trials to assess the efficacy of treatment options. (Also addresses behavioral and systemic dimensions; see above and below).

Malych et al. provide new insights into the cytopathogenicity of *N. fowleri* through original research utilizing biochemical and bioimaging techniques which show changes in the amoeba at the molecular level after host infection as well as virulence factors that allow the pathogen to evade host immune response [26]. These findings reveal potential targets for therapeutic agents.

Siddiqui et al. (2025) review emerging therapies, including nanoparticle-based drugs and intranasal delivery methods, but note that the lack of a large patient population precludes clinical trial design [28]. The authors emphasize the need for early detection and suggest increased surveillance and novel disinfection methods in drinking water distribution systems, also noting that a changing climate may necessitate adaptability in public health and clinical response. (Also addresses environmental and infrastructural dimensions; see above and below).

Kaszubski et al. review the characteristics, pathogenesis, diagnosis, and treatment of four species of parasites (one of which is *N. fowleri*) that may be expanding their natural habitats due to warming temperatures, changes in precipitation, or other climatic factors [37]. They call for increased vigilance among healthcare workers to effectively combat these diseases. (Also addresses environmental dimension; see above).

### 4.8. Infrastructural Risk Dimension

Two articles were coded as primarily addressing infrastructural risk. Risk factors included surveillance capacity in water distribution systems and susceptibility of the organism to disinfecting agents at high temperatures. As viewed through the lens of the IPCC framework, this risk dimension corresponds most directly to the concept of *exposure*.

Furst et al. review effects of rising temperatures on drinking water distribution systems, with a focus on pathogens, including *N. fowleri* [34]. They note that thermotolerant pathogens can be controlled with chlorine or chloramine, but high temperatures degrade disinfectants, especially chlorine, reducing protection. Additionally, they found drinking water distribution systems can reach temperatures up to 52 °C, risking the survival of pathogens due to degraded disinfectant. Gaps in knowledge are identified regarding the effect of high temperatures on drinking water quality, disinfectant decay, and opportunistic pathogens.

Ward and Sherchan report on finding *N. fowleri* in both raw water sources and water distribution systems in Louisiana [33]. They emphasize the importance of monitoring residual chlorine at the end of distribution lines. Furthermore, they note that the impact of a warming climate on *N. fowleri* in water distribution systems is understudied.

## 5. Discussion

This scoping review maps the extent and nature of the peer-reviewed literature between 2012 and 2025 addressing the risk of *N. fowleri* infection in the context of climate change. Twenty-seven articles met inclusion criteria. Of these, only nine framed research directly within the context of climate change; the other 18 mentioned climate change as a possible factor affecting outcomes related to the amoeba.

Analysis of the 27 articles revealed four dimensions of risk—environmental, behavioral, clinical/biological, and infrastructural—that emerged inductively rather than from a predetermined framework (See Supplementary Table S1). The environmental risk dimension, with nine articles, highlighted the need for further study of possible expansion of the geographic range of *N. fowleri* related to a warming climate; biotic and abiotic factors that encourage growth such as temperature, pH, salinity, and/or presence of other microbes in the habitat; and the need for accurate ecological niche modeling for predicting future risk. The behavioral risk dimension, with two articles, explored factors that bring humans into contact with *N. fowleri*, such as recreational water exposure, sinus rinsing, and travel. What remains to be studied is not only the effect warming temperatures will have on the pathogen’s expanded geographic range but also how warming temperatures will influence human engagement in recreational water activities and who is most at risk of contracting PAM. The clinical/biological risk dimension included the most articles (14) and highlighted the need for standards in efficient diagnosis (because the diagnosis is often definitively determined post-mortem) and treatment, and further research into the organism’s pathogenicity and virulence factors. Because of the rarity of PAM, clinical trials are not possible, making the translation of in vitro protocols to effective therapeutic agents difficult. Additionally, risk is increased in under-resourced settings [30]. It is emphasized that PAM should be on every clinician’s differential diagnosis, regardless of latitude, when a patient has symptoms and history suggestive of exposure to *N. fowleri.* The infrastructural risk dimension, with two articles, underlines a gap in surveillance and disinfection methods, including biotic and abiotic factors that encourage growth, and the decreased efficacy of chlorination at high temperatures.

Overall, perhaps the largest gap in knowledge regarding *N. fowleri* and PAM incidence is the true global burden of disease. As stated earlier, historically, PAM has been underreported due to misdiagnosis as bacterial or viral meningitis [1]. Though the numbers of reported cases of PAM have increased globally since the year 2000, it remains unclear whether this observation reflects a true increase in disease burden or if it is attributable to greater awareness among clinicians and/or more advanced diagnostic methods and tools. Nevertheless, because of infection incidence outside of its expected geographic range, *N. fowleri* can be considered a potentially emerging, climate-sensitive pathogen.

**Figure 2:**
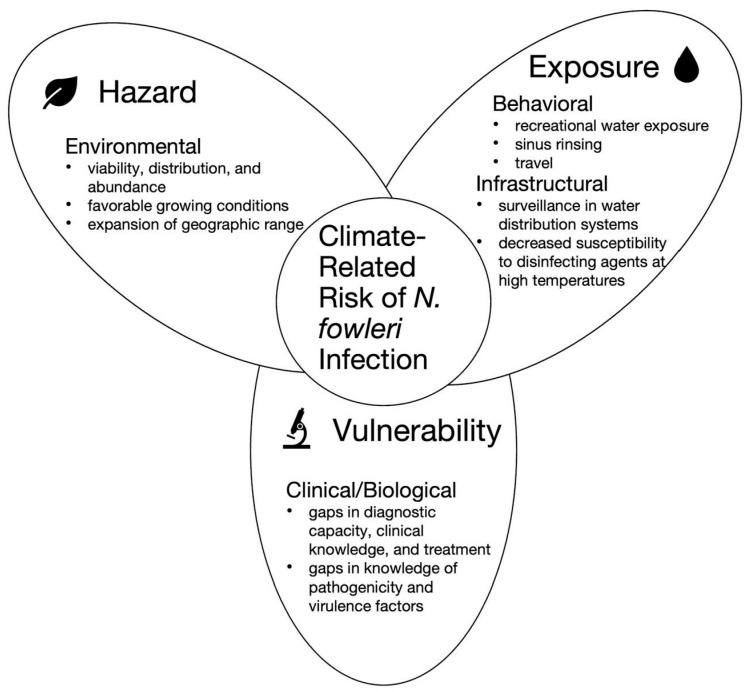
Mapping of Risk Dimensions onto the IPCC AR6 Risk Framework. Inductively derived risk dimensions were mapped onto Intergovernmental Panel on Climate Change AR6 (IPCC AR6) risk framework.

In order to better illuminate the risks and gaps, and to situate findings of this review within a broader theoretical scheme, the dimensions of risk that emerged above can be mapped onto the IPCC AR6 risk framework, in which risk involves the interplay of hazard, exposure, and vulnerability (see Introduction and Figure 2). First, the environmental dimension maps to the concept of *hazard*, which is defined as “the potential occurrence of a natural or human-induced physical event or trend that may cause loss of life, injury, or other health impacts….” [18]. This is the climate threat itself. *N. fowleri* is a thermophilic pathogen that causes the swiftly deadly PAM. Evidence suggests poleward expansion of habitat due to rising global temperatures [4,25,31]. Second, both the behavioral and infrastructural dimensions correspond to the concept of *exposure*, defined as “the presence of people; livelihoods; species or ecosystems; environmental functions, services, and resources; infrastructure; or economic, social, or cultural assets in places and settings that could be adversely affected.” [18]. This is where humans come into contact with the hazard through recreational water exposure, sinus rinsing, and travel, or water distribution systems. Lastly, the clinical/biological dimension maps to the concept of *vulnerability*, which is defined as “the propensity or predisposition to be adversely affected. Vulnerability encompasses a variety of concepts and elements, including sensitivity or susceptibility to harm and lack of capacity to cope and adapt.” [18]. These are the conditions that determine the extent of harm resulting from exposure. As mentioned above, standards are lacking in efficient diagnosis and treatment. The rarity of PAM makes clinical trials for novel therapeutic agents impossible. Furthermore, under-resourced settings that lack capacity are particularly vulnerable to the effects of this and other climate-related health hazards.

Through the IPCC framework lens, we can see a risk profile begin to take shape. The hazard (*N. fowleri*) is well considered with evidence pointing to an expanding geographical range, but neither the biotic/abiotic factors that encourage its growth nor its changing ecological niche in the context of climate change are well defined. Exposure brings us to the interface of humans with the hazard and questions remain unanswered about how human behavior may change and how best to adapt drinking water distribution systems to disable the hazard in the context of a warming climate. Finally, vulnerability is where we see the greatest constraints due to lack of knowledge regarding how best to diagnose and treat after humans are exposed to the hazard. Without major breakthroughs in the area of research regarding vulnerability, the best goal is prevention.

Implications for public health preparedness can be organized according to the standard three levels of prevention—primary, secondary, and tertiary [41]. Starting with primary prevention, the level at which the aim is to prevent the onset of disease or other adverse events before they arise, priorities should focus on systematic surveillance of recreational waters and water distribution systems, public awareness campaigns, including multi-lingual, accessible signage displayed at recreational waters, public availability of nose clips where conditions may be hospitable for the growth of *N. fowleri,* and routine clinician guidance on safe water recreation practices to those most at risk [1,16]. Furthermore, education about sterile nasal sinus rinsing practices should be made available in appropriate situations.

Secondary prevention encompasses screening and early detection. The first goal here is to raise awareness among clinicians and public health practitioners [4,17,20,25]. *N. fowleri* infection should be on every clinician’s differential diagnosis when symptoms and history are consistent with exposure to the pathogen, and treatment should begin empirically. Molecular diagnostic tools such as metagenomics and PCR sequencing of cerebral spinal fluid show promise in providing early diagnosis [30].

Tertiary prevention, the level at which harm is mitigated once disease takes root, is a challenge at this time, given the lack of standardized treatment protocols, though some research has shown promise of progress [17,21,22,26,28,29,38,39].

At this time, public health prevention goals can be prioritized at the first two levels—primary and secondary—with primary prevention focused on characterizing the *hazard* through environmental testing and determination of geographic range, and secondary prevention specifically focused on reducing *vulnerability* via encouraging clinician awareness of PAM, inclusive of patient history and disease symptoms. As we do not yet know the true burden of disease, centering solely on primary prevention efforts through non-targeted public messaging, though well-intended, could cause disproportional alarm regarding an exceedingly rare disease. Clinical suspicion is the ultimate hurdle that must be overcome in order to eschew misdiagnosis, pursue proper screening (CSF analysis sent to specialized laboratories), initiate early empiric treatment, and quantify true disease burden. Given resource constraints, focus should be on (1) characterizing the *hazard*, and (2) addressing *vulnerability* by encouraging clinician awareness, which serves double duty in both initiating early empiric treatment and efforts to quantify true disease burden.

### Limitations

This review has several limitations. First, as a single-author review, screening and data extraction were not independently verified and inter-rater reliability was not calculated. Second, the risk dimensions were derived inductively. A different coding scheme might yield a different analysis. Third, only three databases were searched—PubMed, Scopus, and Environment Complete. Gray literature and non-English records were not included. Fourth, the search was conducted through December 2025. Any records published after that date were not included. Fifth, this review was not designed as a broad survey of the literature on *N. fowleri*. Search terms were deliberately restricted to situate findings within the context of climate change. A broad search may yield different results.

## 6. Conclusions

This scoping review mapped and synthesized the peer-reviewed literature (2012–2025) published on the risk of disease caused by *N. fowleri* in the context of climate change. Four key dimensions of risk—environmental, behavioral, clinical/biological, and infrastructural—were identified. Research gaps in the IPCC AR6 domains of hazard, exposure, and vulnerability were also identified. The *hazard* (*N. fowleri* and environmental risk) is well considered, but neither the factors that encourage growth nor its changing ecological niche are well defined. *Exposure* (behavioral and infrastructural risk) is underquantified, and questions remain about how human behavior may change and how best to adapt drinking water infrastructure to disable the hazard. *Vulnerability* (clinical/biological risk) presents the greatest constraints due to gaps in diagnostic and treatment capacity after humans are exposed to the hazard. Finally, future directions for public health preparedness were organized according to primary, secondary, and tertiary levels of prevention. Emphasis should be placed on characterizing the *hazard* through environmental testing and determining geographic range, and addressing *vulnerability* by increasing clinician awareness, which serves double duty in both initiating early empiric treatment and efforts to quantify true disease burden.

## Data Availability

Data is contained within the article or Supplementary Materials.

## Supplementary Materials

The following supporting information can be downloaded at: https://www.mdpi.com/article/10.3390/ijerph23060764/s1, Table S1: Articles, Findings, and Knowledge Gaps Mapped onto the IPCC AR6 Hazard, Exposure, and Vulnerability Framework. Table S2: Preferred Reporting Items for Systematic reviews and Meta-Analyses extension for Scoping Reviews (PRISMA-ScR) Checklist [42].
