# Supplementary material for: Naegleria fowleri and Risk of Primary Amoebic Meningoencephalitis in a Changing Climate: A Scoping Review of Biomedical Literature
Source: Int J Environ Res Public Health. 2026 Jun 6;23(6):764. doi: 10.3390/ijerph23060764 (PMC13299504; doi:10.3390/ijerph23060764)
Supplement: Supplementary file 1 [file ijerph-23-00764-s001.zip › ijerph-4328006-supplementary-Table S1.pdf]

Table S1. Articles, Findings, and Knowledge Gaps Mapped onto the IPCC AR6 Hazard, Exposure, and Vulnerability Framework

Hazard	Exposure		Vulnerability
**Environmental**	**Behavioral**	**Infrastructural**	**Clinical / Biological**
Bright 2017 - Several cases of PAM have been linked to groundwater and geothermally heated waters; *N. fowleri* occurs naturally in groundwater and geothermally heated waters; Gaps: "why the organism persists in some water sources and not in others is not well understood. The role of biofilms in groundwater wells and plumbing in individual buildings, and the potential for warming due to climate change to expand the occurrence of the organism into new regions, are still unclear."	Diaz 2012 - Prevention via education and behavior modification: avoiding water-related activities in bodies of warm freshwater (including near electrical power plants), avoiding getting water up nose, avoid stirring up sediment, use properly treated water in neti pots for sinus rinsing; risk is highest for young men; Gap: more studies needed regarding increased risk in warming climate	Furst 2024 - Thermotolerant pathogens can be controlled with chlorine or chloramine, but high temps degrade disinfectants (esp. chlorine) reducing protection. found drinking water systems reach temps up to 52 degrees celsius, risking survival of pathogens due to degraded disinfectant. Gaps in knowledge regarding effect of high temperatures on drinking water quality, disinfectant decay, and opportunistic pathogens	Cooper 2019 - Geographic range may be expanding; new therapies including neuron-saving techniques (dexamethasone, CSF drainage, induced hypothermia) may result in cure if diagnosed early.
Dey 2023 - Evidence of *Naegleria spp.* in Canadian water (no *Naegleria fowleri* found), suggestive of northward expansive range of *Naegleria spp.,* ongoing water monitoring and management is suggested Gap: knowledge of	Hong 2023 - First case in South Korea (imported from Thailand); importance of including PAM in differential diagnosis for severe meningoencephalitis, esp after travel to tropical regions	Ward 2023 - *N. fowleri* found in both "raw source water" and water distribution systems in Louisiana; importance of monitoring residual chlorine at the end of distribution lines: Gap: impact of changing climate	Hall 2024 - Characteristics and treatment course of 5 survivors of PAM; argue PAM diagnosis is largely dependent on patient's history of exposure, physician's high index of suspicion, and CSF studies. Points out gap in experimental trials

distribution and microbial ecology			to assess efficacy of treatment options
Kemble 2012 - Northernmost case of PAM in the US (Minnesota); first raised question of possibility of expanded geographic range; suggest communication with public and clinicians regarding risk; suggestion that other factors such as intra-amebic bacteria could alter pathogenicity or host immune response			Herman 2021 - Identification of possible novel pathogenicity factors via genomics and transcriptomics, comparison to non-pathogenic *Naegleria spp.*; possible therapeutic targets
Leal dos Santos 2022 - Incidence of PAM increasing; new treatments that do not contribute to antimicrobial resistance needed; further investigation of how human-caused ecosystem changes lead to dispersion of pathogen			Kaszubski 2025 - Focus on four species of parasites (one is *N. fowleri*), possible expansion of habitat due to climate change, possible increased incidence of disease.
Maciver 2020 - Gaps: "What is the true burden of PAM worldwide? Is the incidence of PAM actually increasing or are we just more aware of it? Is the geographic distribution expanding and/or will it, due to climate change? Why do young males seem to be at more risk than			Kou 2025 - Combined metagenomic next-generation sequencing (mNGS), targeted PCR, and microscopic analysis of CSF may be tools for rapid molecular diagnostics; suggests clinicians empirically initiate anti-amoebic therapy to patients with case

other people? What factors dictate the distribution of *N. fowleri*? How will we diagnose cases more rapidly, and how can we treat these cases more effectively?"			presentation and history consistent with infection with *N. fowleri*; suggests public health efforts center on monitoring, disinfecting recreational water facilities, and alerting public to high-risk periods
Stahl 2020 - Gap: Efficient *N. fowleri* detection and quantification method needed for study of microbial ecology and abiotic and biotic factors that affect its growth and proliferation			Malych 2025 - Changes in *N. fowleri* at the molecular level after host infection; virulence factors; identification of possible therapeutic targets
Stahl 2023 - *N. fowleri* was found to grow best at 25 degrees C; growth driven by temperature and salinity (lower temperature encouraged growth in high salinity environments, while lower salinity consistently encouraged growth at all temperatures)			Mungroo 2022 - Infections on the rise; need for less invasive, faster, more sensitive diagnostic techniques; limited, non-standardized treatment options. Further research into: a need for expanding in vitro research into viable therapies, more research into metal-conjugated nanoparticles and intra-nasal drug delivery for therapy, and an urgent need for interdisciplinary research collaborations between academia, the pharmaceutical industry, and water

			distribution utilities.
Stahl 2025 - "Differences between future and present-day predictions showed that increases in habitat suitability for some geographic regions, especially in northern states, suggested a potential northward expansion of *N. fowleri*. Future research should experimentally test associations between environmental variables and the presence, survival, and proliferation of *N. fowleri* to further refine ecological niche models for these pathogenic amoebae."			Phung 2025 - Vietnamese case (10-month-old), no direct exposure to untreated freshwater reported, multiplex real-time PCR in underresourced area for diagnosis
Xue 2018 - *N. fowleri* found in brackish water; water temperature and concentration of *E. coli* "indicative" of *N. fowleri* concentration; more research needed on role of sediment as source of *N. fowleri* in water column			Rizo-Liendo 2019 - Fluvastatin as potential treatment for PAM; call for further studies into the efficacy of statins as a therapeutic agent against PAM
			Siddiqui 2022 - Medicinal plant compounds had amoebicidal activity against both *N. fowleri* and *B. mandrillaris*; possible

			treatment/water disinfectant capabilities
			Siddiqui 2022 - In vitro, imidazothiazole compounds shown to have amoebicidal properties against *N. fowleri*, while exhibiting minimal cytotoxicity and decreasing amoeba-meditated cytotoxicity to human cells; need to determine mechanism of action via electron microscopy; suggest testing with cyst form; suggest further testing in vivo with animal models
			Siddiqui 2022 - Various compounds conjugated with zinc oxide nanparticles showed antiamoebic properties, while showing little cytotoxicity to human cells; Gap: testing compounds against cyst form; studies on using ZnO conjugates in water storage for disinfectant effects
			Siddiqui 2025 - Emerging therapies (including nanoparticle-based drugs) and mode of delivery (intranasal), but lack of large patient

			population precludes clinical trial design; suggest increased surveillance and novel disinfection methods in drinking water distribution systems; emphasizes need for early detection; changing climate may necessitate adaptability in our response
			Zhang 2021 - Gap: standards for diagnosis and treatment of encephalitis caused by FLA
